# *Endothelin Receptor B2* (*EDNRB2*) Is Responsible for the Tyrosinase-Independent Recessive White (*mo^w^*) and Mottled (mo) Plumage Phenotypes in the Chicken

**DOI:** 10.1371/journal.pone.0086361

**Published:** 2014-01-23

**Authors:** Keiji Kinoshita, Toyoko Akiyama, Makoto Mizutani, Ai Shinomiya, Akira Ishikawa, Hassan Hassan Younis, Masaoki Tsudzuki, Takao Namikawa, Yoichi Matsuda

**Affiliations:** 1 Avian Bioscience Research Center, Graduate School of Bioagricultural Sciences, Nagoya University, Nagoya, Japan; 2 Department of Biology, Keio University, Yokohama, Japan; 3 Laboratory of Animal Genetics, Department of Applied Molecular Biosciences, Graduate School of Bioagricultural Sciences, Nagoya University, Nagoya, Japan; 4 Department of Poultry Production, Faculty of Agriculture, Kafr El-Sheikh University, Kafr El-Sheikh, Egypt; 5 Laboratory of Animal Breeding and Genetics, Graduate School of Biosphere Science, Hiroshima University, Higashi-Hiroshima, Japan; 6 Japanese Avian Bioresource Project Research Center, Hiroshima University, Higashi-Hiroshima, Japan; University of Lausanne, Switzerland

## Abstract

A mutation that confers white plumage with black eyes was identified in the Minohiki breed of Japanese native chicken (*Gallus gallus domesticus*). The white plumage, with a few partially pigmented feathers, was not associated with the tyrosinase gene, and displayed an autosomal recessive mode of inheritance against the pigmented phenotype. All F_1_ offspring derived from crosses with mottled chickens (*mo*/*mo*), which show characteristic pigmented feathers with white tips, had plumage with a mottled-like pattern. This result indicates that the white plumage mutation is a novel allele at the *mo* locus; we propose the gene symbol *mo^w^* for this mutant allele. Furthermore, the F_1_ hybrid between the *mo^w^*/*mo^w^* chicken and the *panda* (*s*/*s*) mutant of Japanese quail (*Coturnix japonica*), whose causative gene is the *endothelin receptor B2* (*EDNRB2*) gene, showed a *mo^w^/mo^w^* chicken-like plumage, suggesting the possibility that the mutations in parental species are alleles of the same gene, *EDNRB2*. Nucleotide sequencing of the entire coding region of *EDNRB2* revealed a non-synonymous G1008T substitution, which causes Cys244Phe amino acid substitution in exon 5 (which is part of the extracellular loop between the putative fourth and fifth transmembrane domains of EDNRB2) in the mutant chicken. This Cys244Phe mutation was also present in individuals of four Japanese breeds with white plumage. We also identified a non-synonymous substitution leading to Arg332His substitution that was responsible for the mottled (*mo*/*mo*) plumage phenotype. These results suggest that the EDN3 (*endothelin 3*)–EDNRB2 signaling is essential for normal pigmentation in birds, and that the mutations of *EDNRB2* may cause defective binding of the protein with endothelins, which interferes with melanocyte differentiation, proliferation, and migration.

## Introduction

Animals with pigment disorders are easily distinguishable from other individuals by virtue of their different body colours. Such disorders have been well analysed at the molecular level, with more than 200 genes associated with pigmentation are known in mammals [1], [2], and the genes associated with plumage colour mutations have also been increasingly identified in birds [3]. Some of these genes or pigments play important roles not only in integumental pigmentation but also in the senses of vision or hearing [1], [4], [5], [6]. The occurrence of novel pigment-disorder mutants provides the opportunity to understand the functions and network of pigmentation-related genes, and to establish whether they might exhibit the same function in multiple species. In this study, we detected white-plumage mutants in a line of Minohiki (MH), a Japanese native breed of chicken (*Gallus gallus domesticus*), and investigated the gene responsible for the pigmentation phenotype.

In the chicken, two autosomal loci associated with white plumage colour are well known. One is an autosomal “dominant white” locus (*I*) that comprises a multiple-allelic series of the gene: dominant white (*I*), smoky (*I^S^*), dun (*I^D^*), and wild type (*i* ^+^) [7], [8]. The dominant white plumage phenotype is often expressed in commercial chicken breeds, as represented by White Leghorn, and *I* is incompletely dominant to the other *I* alleles. Genetic linkage mapping and nucleotide sequencing revealed that the responsible gene for the dominant white plumage is *PMEL17*, which encodes a matrix protein of pre-melanosomes [9]. The other locus is an autosomal “recessive white” locus (*c*), which comprises recessive white (*c*), albino (*c^a^*), red-eyed white (*c^re^*), and wild-type (*C* ^+^) alleles [8], [10]. The former three allelic genes (*c*, *c^a^*, and *c^re^*) are associated with exclusively white plumage, whereas the wild type (*C* ^+^) has pigmented plumage. These pigmentation phenotypes at the *c* locus are caused by multiple alleles of the tyrosinase (*TYR*) gene, which encodes a key enzyme required for melanin synthesis [11], [12], [13], [14]. White plumage in chickens is considered to be mostly controlled by these two loci, although allelism between different breeds with white plumage has not been sufficiently tested to confirm the identity of the locus responsible for white plumage.

On the other hand, the molecular basis of white spotting coat phenotypes, in which the ability of cells on particular parts of the skin to make pigment is impaired and the skin becomes pink and the fur white, have been well characterised in rat and mouse. This pattern is caused by abnormalities in the differentiation, survival, and migration of melanocytes derived from neural crest cells [15], [16], [17], [18]. Abnormality of the *endothelin* (*EDN*) and *endothelin receptor B* (*EDNRB*) genes are well known to cause the white spotting phenotype. Endothelins (EDNs), which exists as the isotypes EDN1, EDN2, and EDN3, are the 21-amino-acid ligands of endothelin receptors EDNRs, which are G protein-coupled receptors. Endothelins were first reported to be vasoconstricting peptides but now are known to have important roles in the proliferation and differentiation of pigment cells not only in the integument but also in internal organs [19], [20], [21], [22], [23], [24], [25], [26]. Endothelins affect the development of neural crest-derived pigment cells in the melanocyte population in a dose-dependent manner *in vitro* in mouse and quail embryos [27], [28], [29], [30], [31].

The two subtypes of EDNRs in human and mouse are EDNRA and EDNRB [18], [20], [32], [33], [34], [35]. EDNRB contains seven transmembrane domains, and is required for the development of melanocytes and enteric neurons [36], [37], [38]. Spontaneous mutations in *EDN3* and *EDNRB* in mouse are designated *lethal spotting* (*ls*) and *piebald lethal* (*s^l^*), respectively. Both homozygous mutants display similar phenotypes of hypopigmentation and aganglionic megacolon [36], [39]. The hypopigmented phenotype is caused by a decrease in the number of melanoblasts and abnormal cell migration [40], [41].

Birds also have the *EDNRB2* gene, a paralog of *EDNRB*, which is conserved in *Xenopus* and platypus but not in zebrafish and therians, such as human and mouse [42], [43]. The avian EDNRB and EDNRB2 show similar affinities for EDN1, EDN2, and EDN3 with no clear pharmacological differences, and do not show differential inhibition by antagonists. The interaction between EDN3 and EDNRB2 is essential for development and migration of neural crest-derived cell lineages [36], [39], [44]. Whereas *EDN3* is expressed in the ectoderm and gut mesenchyme [45], *EDNRB2* is expressed throughout the melanocyte lineage [42]. In Aves, EDNRB2 is important for melanoblast migration along the dorsolateral pathway [46], [47]. In Japanese quail (*Coturnix japonica*), mutation of *EDNRB2* is associated with the *panda* (*s*) and *dotted-white* (*s^dw^*) plumage phenotypes. The *panda* mutant has white plumage with coloured patches on the head, back, tail, cheek, and wings, whereas the *dotted-white* mutant has a large area of white plumage more than the panda mutant, with only a few small coloured spots on the head and/or back and occasionally with no coloured spots [48], [49], [50]. These mutant plumage phenotypes of Japanese quail are similar to those of the mottled chicken and white MH lines that were the focus of the present study.

Minohiki is a Japanese long-tailed chicken breed, and was designated a national natural treasure of Japan in 1940. The MH breed has been maintained as a closed colony at Nagoya University, Japan, since 1988. Typically, MH individuals are covered with light brown plumage either with or without the Columbian (*Co*) plumage pattern, in which black pigment is restricted to the hackle, wing, foot and tail feathers, but often display a white plumage colour mutation with a few partially pigmented feathers on the hackle, shoulder, or tail. Neonatal chicks with the white plumage mutation have whitish yellow down and often exhibit a small brown spot on the head. In a preliminary crossing test of white MH with white Ukokkei (Japanese Silkie) that was homozygous for a recessive *c* allele, all F_1_ hybrids had coloured (normally pigmented) plumage. This finding strongly suggests that the white plumage mutation of MH is controlled by a novel *TYR* (*c*)-independent autosomal recessive gene. Mottling (*mo*) is a common plumage colour-pattern mutation, in which pigmented feathers have white tips, in many chicken breeds worldwide. The chick down phenotype of the white MH mutant is similar to that of the mottled phenotype, although a mottled chicken develops pigmented feathers with a white tip in the adult. This “mottling” pattern is controlled by an autosomal recessive gene designated *mo* [51], [52], [53]. In addition, another allele at the *mo* locus, the pied plumage represented by Exchequer Leghorn, often exhibits mottled-like feathers and infrequently all black, all white, or mixed feathers [53]. In the current study, we performed allelism tests and linkage mapping to identify the candidate gene associated with the novel white plumage and mottled plumage, and determined nucleotide sequences of the candidate gene for the novel white and mottled plumage patterns in several other chicken breeds. We present strong evidence that *EDNRB2* is associated with these mutant plumage patterns in chickens.

## Results

### Characteristics of the white plumage mutant

**Figure 1:**
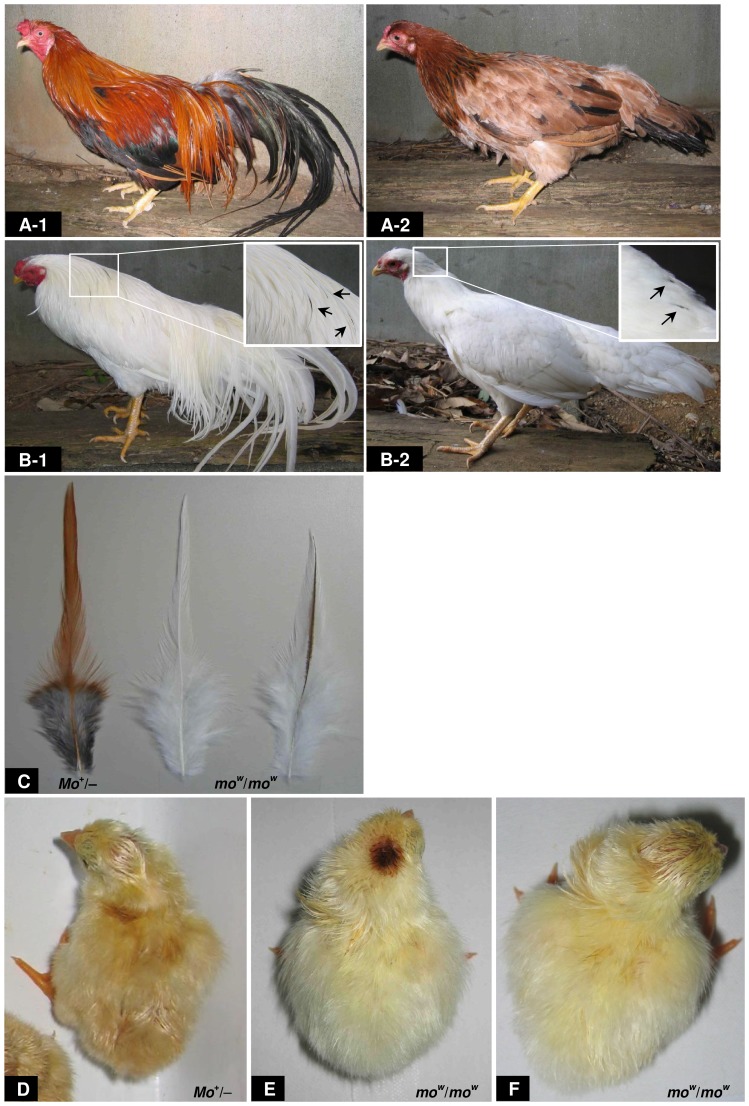
Plumage phenotypes in the Minohiki (MH) line. (A, B) Plumage colours in adult MH chickens with wild-type plumage (*Mo* ^+^) (*i^+^*/*i^+^*, *Co*/*−* or *co* ^+^/*co* ^+^, *C* ^+^/*C* ^+^) (A-1, A-2) and white plumage (*mo^w^*/*mo^w^*) (B-1, B-2). Males are shown in A-1 and B-1, and females are shown in A-2 and B-2. The white mutant shows exclusively white plumage, with the exception of a few partially pigmented feathers at the back of the head, neck, and/or saddle hackle. Squares show magnified areas. Arrows indicate partially pigmented feathers. (C) Feathers of the neck hackle from a wild-type MH adult male (*Mo* ^+/−^) (left) and white MH adult male (*mo^w^*/*mo^w^*) (middle and right). (D–F) Down colours of newly hatched chicks of the wild type (*Mo* ^+/−^) (D) and white mutant (*mo^w^*/*mo^w^*) (E, F). The white mutant chick (*mo^w^*/*mo^w^*) has yellowish down with either a pigmented spot (E) or no spot (F) on the head.

**Figure 2:**
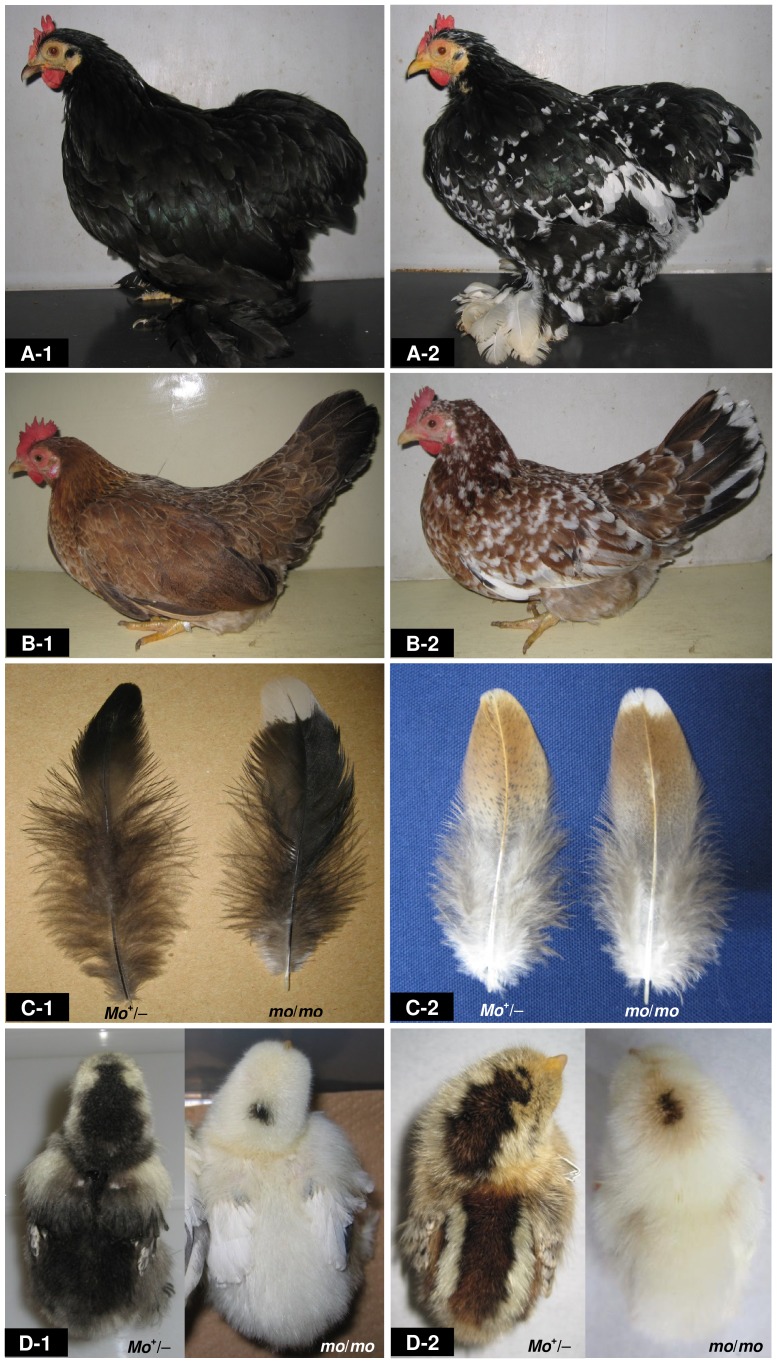
Wild-type and mottled plumage phenotypes with two different *extended black* (*E*) backgrounds in Cochin bantam (CB) and Ehime-jidori (EJ). (A) The wild-type (*Mo* ^+/−^) (A-1) and mottled plumage (*mo*/*mo*) (A-2) in adult CB females with an *E*/*E* genetic background. (B) The wild-type (*Mo* ^+/−^) (B-1) and mottled plumage (*mo*/*mo*) (B-2) in adult EJ females with an *e* ^+^/*e^+^* genetic background. (C-1) Feathers of the saddle from the wild-type CB female (*Mo* ^+/−^) (left) and the mottled CB female (*mo*/*mo*) (right). (C-2) Feathers of the saddle from the wild-type EJ female (*Mo* ^+/−^) (left) and the mottled EJ female (*mo*/*mo*) (right). (D) Down colour of newly hatched chicks of the wild type (*Mo* ^+/−^) (D-1, left) and the mottled type (*mo*/*mo*) (D-1, right) in CB, and the wild type (*Mo* ^+/−^) (D-2, left) and the mottled type (*mo*/*mo*) in EJ (D-2, right). These mottled-type chicks (*mo*/*mo*) have white yellowish down with pigmented spots on the head.

The typical plumage of the MH line is light brown either with or without the Columbian plumage (*Co*) pattern (*i* ^+^/*i* ^+^, *e* ^+/−^ or *e^y^*/*e^y^*, *Co*/− or *co* ^+^/*co* ^+^, *C* ^+^/*C* ^+^) (Figures 1A-1 and A-2). Mutant males and females exhibited white plumage over the whole body except for a few partially pigmented feathers on the head, hackle, shoulder, saddle, and tail along the dorsal region (Figures 1B-1 and 1B-2). This plumage pattern was prominent in adult males (Figures 1B-1 and 1C). In adult females, slightly pigmented feathers were located in the dorsal occipital area (Figure 1B-2). Newly hatched chicks of the wild type displayed exclusively brownish-yellow down feathers (Figure 1D), and the white mutant occasionally showed wholly whitish yellow down feathers with one or two small brown spots (Figure 1E) or no spots (Figure 1F) on the head. The pigmented head spot mostly disappeared as the chicks approached maturity. The white mutant individuals appeared healthy and displayed normal fertility and the existence of pigments in the iris pigment epithelium, and the visual and auditory senses were normal.

### Mode of inheritance of the white plumage mutation

To examine the association of the white plumage mutation of the MH line with the dominant white (*I*) locus and/or recessive white (*c*) locus, white mutant MH females were mated with males of two tester lines: PNP/DO (the wild type with normally pigmented plumage, *i* ^+^/*i* ^+^, *C* ^+^/*C* ^+^) and CAL (autosomal recessive albino, *i* ^+^/*i* ^+^, *c^a^*/*c^a^*) (Table 1). In the cross with the PNP/DO line, 26 F_1_ individuals all exhibited wild-type plumage, and 26 F_2_ progeny were segregated into 20 of the wild type and six of the black-eyed white type with a ratio close to 3∶1 (0.80>*P*>0.70). In the cross with the CAL, 15 F_1_ individuals all exhibited wild-type plumage, and the 307 F_2_ progeny were segregated into 184 wild type, 58 black-eyed white, and 65 red-eyed white (albino) phenotypes with a ratio approaching 9∶3∶4 (0.30>*P*>0.20). These results collectively indicated that the white plumage mutation in the MH line was controlled by a *PMEL17* (at *I* locus)- and *TYR* (at *c* locus)-independent autosomal recessive gene.

### Allelism of the white and mottled (*mo*) plumage phenotypes of chickens and the *panda* (*s*) plumage phenotype of Japanese quail

**Figure 3:**
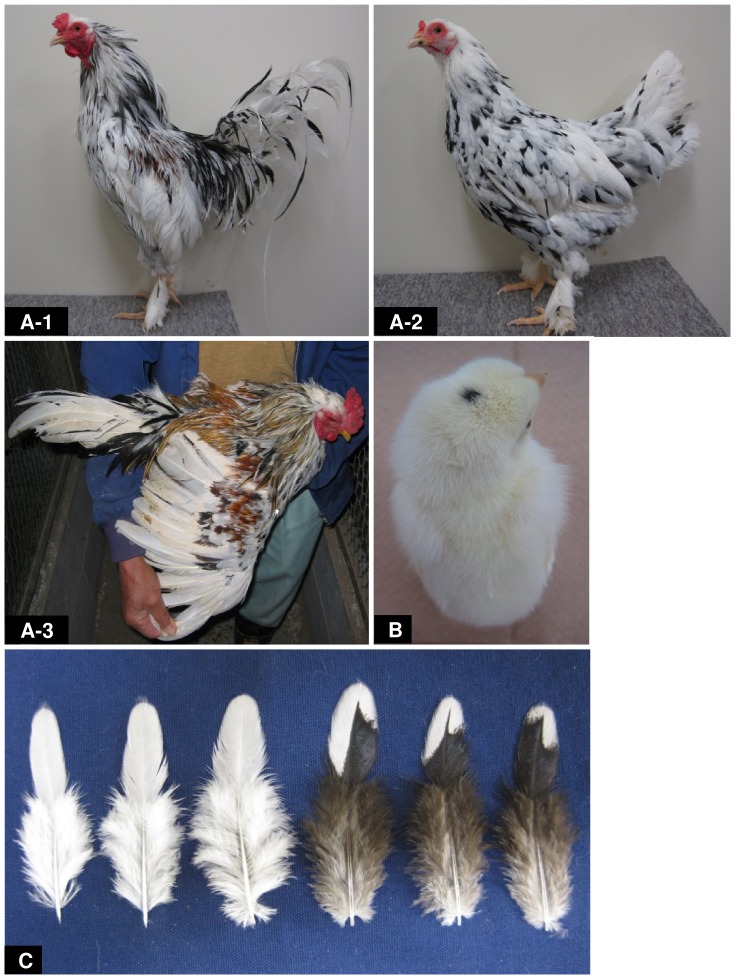
Allelism of mottled (*mo*) and white (*mo^w^*) plumage. (A-1, A-2) An F_1_ adult male (A-1) and an F_1_ adult female (A-2) that were obtained from a mating of a mottled CB male with a white MH female. (A-3) F_1_ adult male that was obtained from mating a mottled EJ male with a white female. All F_1_ adult chickens derived from the two matings exhibited mottled-like plumage. (B) A newly hatched chick obtained from the mating of a mottled CB male with a white MH female, which has whitish down with a pigmented spot on the head. (C) Feathers of the saddle from a F_1_ female (A-2) obtained from the mating of a mottled CB male with a white MH female. In the F_1_ progeny, pure white feathers (left) and black feathers with non-pigmentation at the tips (right) were mixed.

Females with the wild-type and mottled phenotypes are shown in Figures 2A-1 and 2A-2 in the CB line and in Figures 2B-1 and 2B-2 in the EJ line, respectively. The genotypes of the extended black (*E*) locus were *E*/*E* in CB and *e* ^+^/*e* ^+^ in EJ. The mottled individuals commonly exhibited pigmented plumage with white markings at the tips of feathers (Figure 2C-1 right in CB; Figure 2C-2 right in EJ). The mottled chicks in both lines exhibited whitish yellow down with small brown-black spots on the head (Figure 2D-1 right in CB; Figure 2D-2 right in EJ). To test whether the white plumage mutant in the MH line is an allelic variant at the mottled (*mo*) locus, mottled males of CB and EJ were mated with white mutant MH females (Table 1). In the mating with mottled CB, both F_1_ males (Figure 3A-1) and females (Figure 3A-2) exhibited mottled-like plumage, which was intermediate between that of the white MH line and mottled CB. Following mating with mottled EJ, F_1_ males and females exhibited similar plumage intermediate between that of the white MH line and mottled EJ (Figure 3A-3). Four F_1_ chicks of mottled CB showed whitish yellow down with one or two black spots on the head as well as the mottled CB phenotype (Figure 3B; Table 1). The plumage of F_1_ hybrids consisted of pure white or black feathers with white spots at the tips of feathers (Figure 3C). Nine F_1_ chicks of mottled EJ also had whitish yellow down with one or two brown-black spots on the head (Table 1). Two female F_1_ hybrids generated by crossing between mottled CB and the white MH mutant were backcrossed with a male white MH mutant, and 40 backcross progeny were obtained. Eighteen and 22 individuals showed the white mutant plumage and mottled-like plumage, respectively. This segregation ratio was close to the expected 1∶1 ratio (0.70>*P*>0.50). These allelism tests confirmed that the mutations in the parental lines were allelic at the same *mo* locus; therefore, we proposed the new gene symbol *mo^w^* for the white plumage mutation in the MH line.

**Figure 4:**
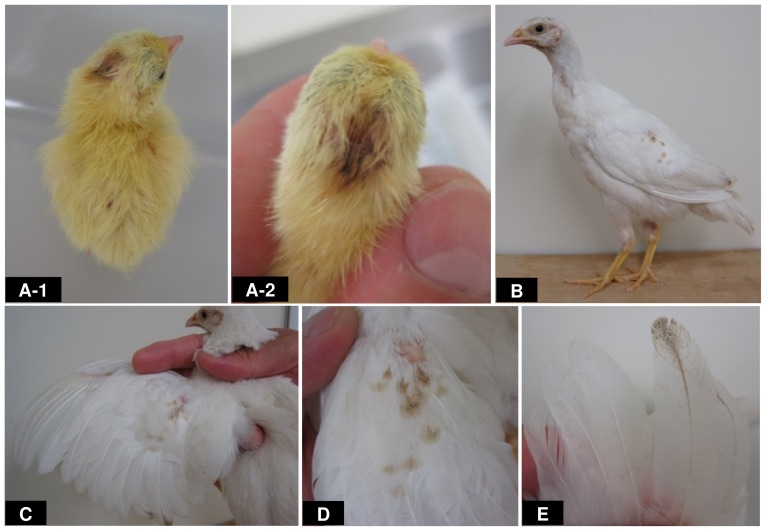
Allelism of white (*mo^w^*) plumage of chicken and panda (*s*) plumage of Japanese quail. (A-1, A-2) A newly hatched F_1_ hybrid chick between the white mutant male of chicken (*mo^w^*/*mo^w^*) and the *panda* mutant female of Japanese quail (*s*/*s*). It has whitish yellow down with one brown spot on the head. (B) The phenotype of 8 week-old young bird of the same individual shown in A-1 and A-2. It has the white plumage with several small coloured patches observed in wings. (C, D) Coloured patches in the wing. (E) Pigmented tip of feather from the wing.

The *panda* mutant of Japanese quail (*s*/*s*) has white plumage with small coloured patches, which is caused by a non-synonymous mutation in the *EDNRB2* gene. We therefore obtained a F_1_ hybrid between the white mutant chicken (*mo^w^*/*mo^w^*) and the *panda* mutant of Japanese quail (*s*/*s*) by artificial insemination. The F_1_ hybrid chick also showed yellowish down with one brown spot on the head as well as the white mutant chicken (Figures 4A-1 and A-2), and several small coloured patches were observed on the wing in the eight week-old bird (Figure 4B–4E, the same individual shown in Figures 4A-1 and A-2). These results show that the mutations in parental species are alleles of the same gene, *EDNRB2*.

### Nucleotide sequences of *EDNRB2* in the *mo^w^* mutant

**Figure 5:**
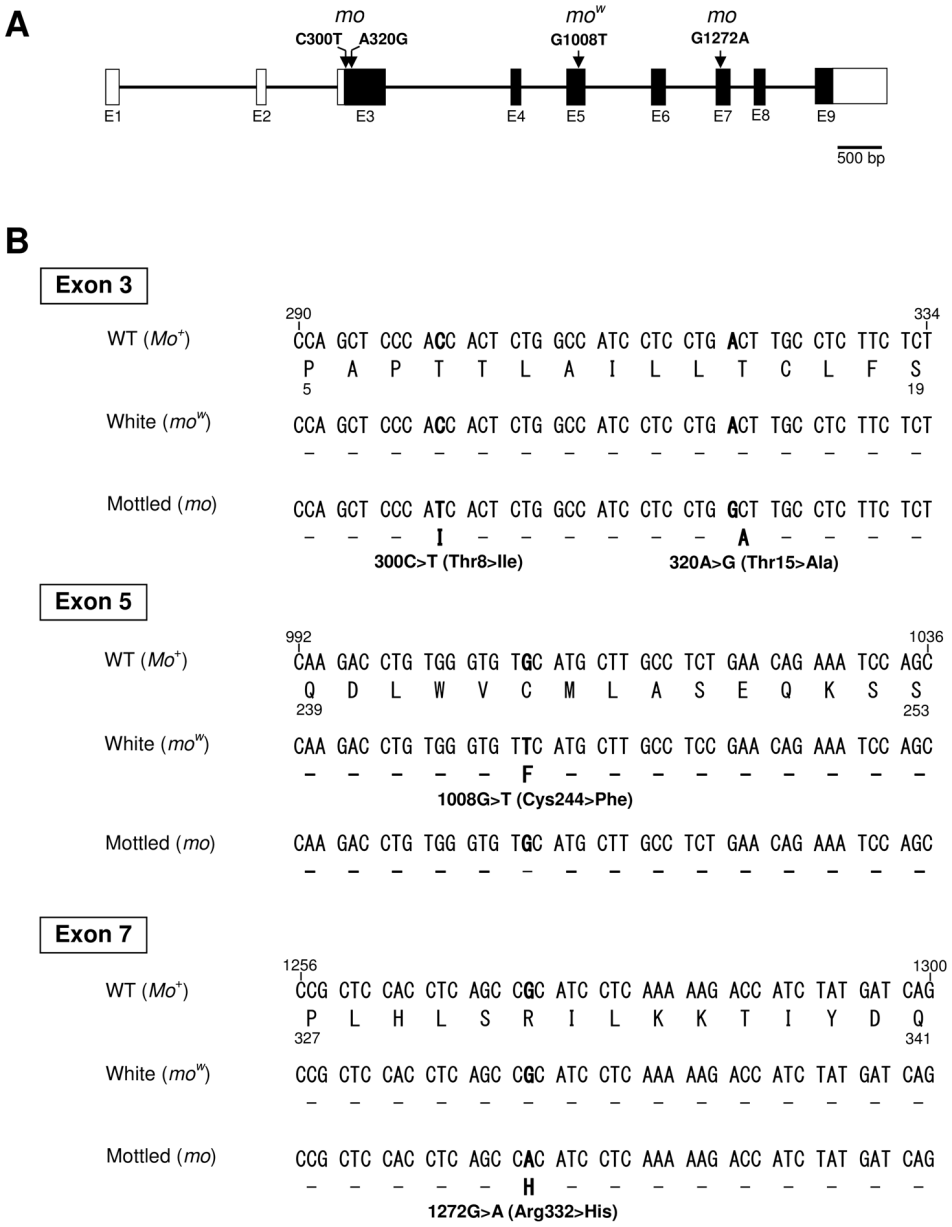
Comparison of *EDNRB2* nucleotide sequences among the wild-type (*Mo* ^+^), white (*mo^w^*), and mottled (*mo*) alleles. (A) A schematic illustration of the genomic structure of chicken *EDNRB2*. Arrows indicate the positions of non-synonymous nucleotide substitutions found in the white mutant (*mo^w^*) and mottled (*mo*) alleles. White boxes indicate untranslated exons at 5′ and 3′ ends, and black boxes indicate the coding exons. (B) Partial DNA sequences of exons 3, 5 and 7 of the wild-type (*Mo* ^+^/*Mo* ^+^), white mutant (*mo^w^*/*mo^w^*) and mottled (*mo*/*mo*) alleles. One non-synonymous substitution (G1008T/Cys244Phe) and three non-synonymous substitutions (C300T/Thr8Ile, A320G/Thr15Ala, and G1272A/Arg332His) are specific to the white mutant (*mo^w^*) and mottled (*mo*) alleles, respectively.

Based on the results of allelism tests, we determined nucleotide sequences of the *EDNRB2* gene in the white MH mutant using the primers shown in Figure S1 and Table S1. Direct sequencing of *EDNRB2* cDNA revealed four single-nucleotide polymorphisms (SNPs) in the 1528 bp fragments between the wild type and the white MH mutant: three synonymous substitutions (C>G at nucleotide position 691, T>C at position 1,021, and T>C at position 1,159) and one non-synonymous G>T substitution at position 1,008. The non-synonymous substitution resulted in an amino acid substitution of cysteine (Cys) by phenylalanine (Phe) at position 244 (Cys244Phe) in EDNRB2 (Figure 5; Table 2). The *EDNRB2* cDNA sequences of the wild type (*Mo* ^+^/*Mo* ^+^) and the white mutant (*mo^w^*/*mo^w^*) were deposited with DDBJ (accession nos. AB697059 and AB697060, respectively). The G1008T substitution (Cys244Phe) was observed in other chicken breeds [Minohiki (Shizuoka), Uzurao, Shokoku, Onagadori, and Ohiki] that show white plumage with a few partially pigmented feathers (Table 3).

### Complete association of G1008T substitution in *EDNRB2* with the *mo^w^* mutation

**Figure 6:**
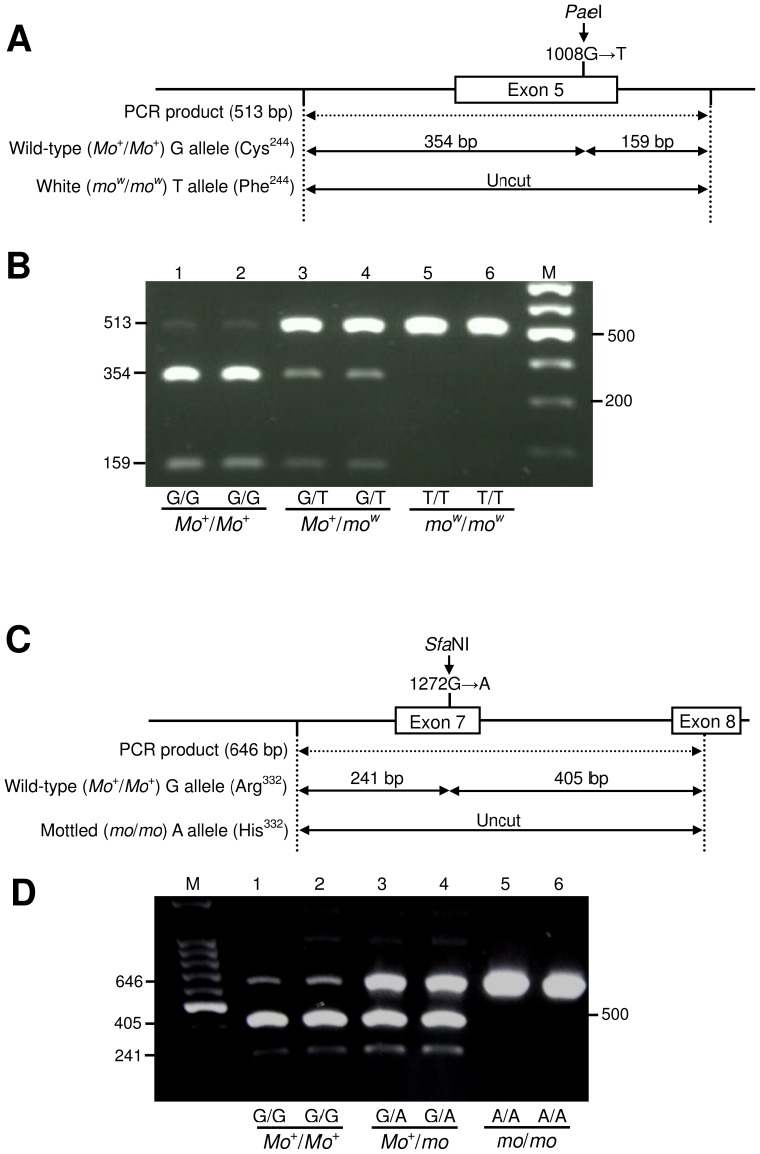
Genotyping of the G1008T (Cys244Phe) mutation in *mo^w^* allele and the G1272A (Arg332His) mutation in *mo* allele. (A) Schematic representation of the *Pae*I-RFLP in the genomic DNA fragment of exon 5 of chicken *EDNRB2*, which was PCR-amplified using the primers EDNRB2_Int4F and EDNRB2_Int5R1 (Figure S1; Table S1) and was used for genotyping of the Cys244Phe mutation. Digestion with *Pae*I (G↑CATG↓C) produces two DNA fragments (354 bp and 159 bp) in the wild-type *Mo* ^+^ allele, whereas the DNA fragments derived from the *mo^w^* allele were not digested with *Pae*I. (B) Genotyping of the F_2_ progeny that were obtained by crossing a CAL male with a white MH female. The genotypes were determined by agarose gel electrophoresis after digestion of the PCR products (513 bp) with *Pae*I. The nucleotides at position 1,008 in six individuals, genotypes, and plumage colour phenotypes are indicated below the lanes. M, Gene Ladder 100 (0.1–2 kbp) (Wako, Japan) was used as a molecular size marker. (C) Schematic diagram of the *Sfa*NI-RFLP in the genomic DNA fragment of exon 7, which was PCR-amplified using the primer set EDNRB2_Int6F and EDNRB2_Ex8R (Figure S1; Table S1) and used for genotyping of the Arg332His mutation. (D) Agarose gel electrophoresis of the PCR products (646 bp) after *Sfa*NI-digestion. Two DNA fragments (241 bp + 405 bp) are produced in the wild-type *Mo* ^+^ allele, whereas the DNA fragments derived from the *mo* allele were not digested with *Sfa*NI. The nucleotides at position 1,272 in six individuals, genotypes, and plumage colour phenotypes are indicated below the lanes. M, Gene Ladder 100 (0.1–2 kbp) (Wako, Japan) was used as a molecular size marker.

To examine the association of *EDNRB2* with the white plumage mutation in the MH line, genotyping of *EDNRB2* was performed for F_2_ progeny obtained from the mating between the white MH mutant and CAL line (Table 1), and their genotypes were compared with the segregation patterns of plumage. The G1008T substitution in exon 5 of *EDNRB2*, which was observed in the white MH mutant, destroyed a *Pae*I recognition site (G↑CATG↓C). Two sizes of fragments (354 bp and 159 bp) were produced by *Pae*I digestion from the wild-type allele (*Mo* ^+^) but not from the white mutant allele (*mo^w^*) (Figure 6A, 6B). The linkage mapping using 93 F_2_ progeny (50 wild-type and 43 white mutant individuals) showed that logarithm of odds (LOD) scores between the *mo* locus and the three markers *ADL0255*, *EDNRB2*, and *MCW0295* were 2.3, 27.2, and 17.1, respectively. No recombination was observed between the T/T genotype (513 bp) of *EDNRB2* and the *mo^w^* allele for the examined 43 white mutant individuals (Figure S2). In the other 149 F_2_ progeny excluding the albino white individuals (*n* = 65), 15 white mutant individuals (*mo^w^*/*mo^w^*) showed the T/T genotype (513 bp), and the remaining 134 pigmented individuals (*Mo* ^+^/*Mo* ^+^ or *Mo* ^+^/*mo^w^*) carried the G/G or G/T genotype (354 bp and 159 bp) (Figure 6B). This result confirmed complete association between the recessive white allele (*mo^w^*) and the G1008T substitution in *EDNRB2*.

### Cys244Phe mutation of *EDNRB2* in different chicken breeds

To confirm that the G1008T (Cys244Phe) substitution in exon 5 of *EDNRB2* causes the white plumage phenotype at the *mo* locus, we genotyped the genomic DNA fragment containing exon 5 of *EDNRB2* for 204 individuals from 22 different breeds, including Red jungle fowl (*Gallus gallus*), using the PCR-RFLP method (Figure 6A, 6B; Table S2). Twelve individuals from the breeds Minohiki (Shizuoka; *n* = 2), Onagadori (Kanagawa; *n* = 4), Ohiki (Hiroshima; *n* = 1), Shokoku (Mie; *n* = 3), and Uzurao (Ehime; *n* = 2) had white plumage with a few pigmented feathers and were homozygous for the T/T allele (Phe/Phe) at position 1,008 (Phe244Phe) as well as the white mutant in MH line (*n* = 8). On the other hand, six individuals with white plumage, two Shokoku (Ehime), four Uzurao (Ehime), which were not homozygous for the T/T allele (Phe/Phe) were confirmed to be homozygous for the recessive white (*c*) locus by a test cross with CAL (*c^a^*/*c^a^*) individuals (data not shown). In addition, the other 38 white-plumaged individuals of six breeds (Chabo, Chan, Koshamo, Japanese Silkie, CB, and White Leghorn) and 140 pigmented-feathered individuals of 22 breeds, including Red jungle fowl, were homozygous for the G allele at position 1,008 with only eight exceptions (six wild-type Minohiki and two Uzurao), which were G/T heterozygotes. These results collectively suggested that the G>T substitution at 1,008 in exon 5, which is responsible for the Cys244Phe substitution, was closely associated with the novel recessive allele (*mo^w^*) showing white plumage with a few pigmented feathers in Japanese native chickens.

### *EDNRB2* mutations at the *mo* locus in mottled chickens

We determined the nucleotide sequences of cDNA fragments of the entire coding region of *EDNRB2* for EJ chickens with the wild-type (*Mo* ^+^/*Mo* ^+^) and mottled (*mo*/*mo*) plumage (AB697061 and AB697062, respectively) (Table 2). Direct sequencing revealed seven SNPs that involved four synonymous substitutions (T508C, C691G, C835T, and T1021C) and three non-synonymous substitutions (C300T, A320G, and G1272A) between the two plumage types. These resulted in amino acid changes of Thr>Ile at position 8, Thr>Ala at position 15, and Arg>His at position 332, respectively (Figure 5; Table 2).

To confirm that the three missense mutations leading to amino acid substitutions (Thr8Ile, Thr15Ala and Arg332His) in *EDNRB2* are associated with the mottled plumage phenotype, we examined the presence of the three mutations in three additional chicken breeds with mottled plumage (CB, Chabo and Uzurao) by genomic sequencing of exons 3 to 9 (Table 3). The three missense mutations were homozygous specifically in the breeds with mottled plumage (EJ, CB, Chabo, and Uzurao). This result indicates that the three non-synonymous nucleotide substitutions are specific for mottled plumage (*mo*/*mo*) and are not associated with white plumage (*mo^w^*/*mo^w^*), although the mottled and white plumage phenotypes resulted from mutations that occurred in the same causative gene. The non-mottled breeds were all homozygous for Thr at position 8, Thr at position 15 and Arg at position 332 except for the Rhode Island Red (RIR-Y8/NU), Sebright bantam and Large Cochin, which had the Thr8Ile and Thr15Ala substitutions. The individuals in RIR-Y8/NU (n = 15) that were homozygous for Thr8Ile and Thr15Ala both showed similar plumage to that of the other non-mottled individuals (n = 3) with Thr at position 8 and Thr at position 15. This suggested that the Arg332His substitution was essential for the mottled plumage phenotype, whereas the two non-synonymous mutations in exon 3 (Thr8Ile and Thr15Ala) might have little effect on plumage colour.

### Arg332His mutation of *EDNRB2* in other chicken breeds

The G1272A (Arg332His) substitution in exon 7 of *EDNRB2* was surveyed for 98 individuals of 14 breeds including Red jungle fowl with a variety of plumage patterns using the PCR-RFLP method (Table S3). Digestion of the 646-bp PCR product with *Sfa*NI produced two fragments (241 bp and 405 bp) for the wild-type allele (*Mo* ^+^) and one uncut fragment (646 bp) for the mottled allele (*mo*) (Figure 6). The 24 individuals of Chabo (Gunma, Hiroshima University, and Hiroshima; *n* = 16) and Koshamo (Kagoshima and Hiroshima University; *n* = 8) with mottled plumage were all homozygous for the A/A allele (His/His) at position 1,272 (His332His), as were mottled EJ (*n* = 8) and CB (*n* = 3) chickens. The other 60 non-mottled individuals of 14 breeds were homozygous for the G allele at position 1,272 except for three non-mottled EJ individuals with heterozygous G/A (Table S3). These results suggested that the G>A substitution at 1,272 in exon 7, leading to the Arg332His substitution, was completely associated with the mottled allele (*mo*) in Japanese native chickens.

### Genomic DNA sequences of *EDNRB2* at the *mo* locus

Nucleotide sequences of the genomic DNA fragments containing the open reading frame from exons 3–9 and introns 3–8 of *EDNRB2* were determined for the wild-type MH, white MH mutant, and mottled EJ chickens using the primers shown in Figure S1, and then compared with the reference genomic sequence on chromosome 4 in the UCSC chicken genome (May 2006 assembly). Seventy-five SNPs were identified among the sequences of the three chicken types compared with the genome database (Table S4). In addition, an 8-bp insertion in intron 3 of MH with the pigmented wild-type plumage (*Mo* ^+^/*Mo* ^+^) and a 4-bp deletion in intron 7 of the white MH mutant (*mo^w^*/*mo^w^*) were observed. The 432-bp LTR/ERVK sequence was located at positions 11,261,544–11,261,975 of intron 5 on the basis of the chicken reference genomic sequence (Type I) (Figure S3), whereas this 432 bp LTR/ERVK sequence was absent from intron 5 in the MH line (*Mo* ^+^/*Mo* ^+^, *mo^w^*/*mo^w^*) and mottled EJ (*mo*/*mo*) (Type II), in which an unrelated 295 bp sequence was present at the same position instead of the 432 bp LTR/ERVK. A homologous sequence was shared with Japanese quail (*Coturnix japonica*) (GenBank accession no. AY360822), which showed 91% nucleotide sequence identity (267/295 bp) (data not shown). This result suggested that the Type II sequence was the ancestral type present in the common ancestor of chicken and Japanese quail, and that this 295 bp sequence element was replaced with the 432-bp LTR/ERVK sequence in an evolutionary lineage of chicken after its divergence.

### Expression of the *mo^w^* and *mo* allelic genes

**Figure 7:**
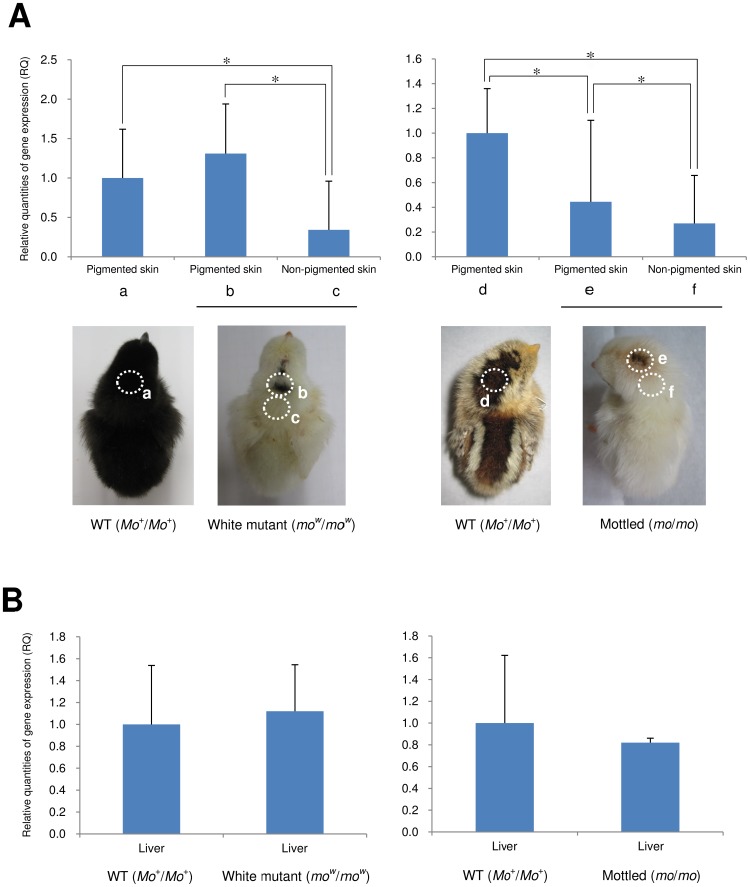
Expression analysis of *EDNRB2* by quantitative real-time RT-PCR in three-day-old chicks with the wild-type (*Mo* ^+^), white (*mo^w^*) and mottled (*mo*) plumage. (A) Relative expression levels in skin tissues of chicks with the wild-type (*Mo* ^+^/*Mo* ^+^) (n = 9) (a) and white mutant (*mo^w^*/*mo^w^*) plumage (n = 6) (b, c) in the F_2_ progeny that were obtained from the mating of a white Shokoku male (*mo^w^*/*mo^w^*) with a GSP female (*Mo* ^+^/*Mo* ^+^); and chicks with the wild-type (*Mo* ^+^/*Mo* ^+^) (n = 3) (d) and mottled type (*mo*/*mo*) plumage (n = 2) (e, f) in EJ line. Dotted circles indicate the regions from which skins were collected for analysis. Each bar shows the average expression level, which is presented as the quantity of gene expression relative to that of the wild type. (B) Expression level in liver tissues of the same individuals which were used for expression analysis of skins: the wild-type (*Mo* ^+^/*Mo* ^+^) (n = 9) and white mutant (*mo^w^*/*mo^w^*) chicks (n = 6) in the F_2_ progeny between GSP and white Shokoku; and the wild-type (*Mo* ^+^/*Mo* ^+^) (n = 3) and mottled (*mo*/*mo*) plumage (n = 2) in EJ. Asterisks indicate a statistically significant difference at *P*<0.05 (one-way ANOVA followed by Tukey HSD test). The vertical bars indicate standard deviations.

The expression levels of *EDNRB2* mRNA in the head skin and liver tissues were examined for three-day-old chicks with the white (*mo^w^*/*mo^w^*) and mottled (*mo*/*mo*) genotypes, and compared with those of the wild type (Figure 7). PCR amplification efficiencies were checked by preparing standard curves using target-specific primers (*EDNRB2*) and control primers (*GAPDH*). PCR amplification efficiencies and correlation coefficient (R^2^) were 90.256% and 0.997 for *EDNRB2* and 92.525% and 0.996 for *GAPDH* (data not shown). The relative expression level of *EDNRB2* in pigmented skin of the white mutant (*mo^w^*/*mo^w^*) was not different from that of the wild-type (*Mo* ^+^/*Mo* ^+^) skin (p>0.05); however, the relative expression level in non-pigmented skin of the *mo^w^*/*mo^w^* chicks was significantly lower than those in pigmented skin of the same *mo^w^*/*mo^w^* individuals and the wild-type skin (1/4 decrease for the non-pigmented skin) (p<0.05) (Figure 7A). In the mottled type (*mo*/*mo*), the *EDNRB2* expression in pigmented skin was significantly lower than the wild-type (*Mo* ^+^/*Mo* ^+^) (p<0.05). The expression was also lower in non-pigmented skin than that in pigmented skin of the same individuals (approximately 1/2 decrease for the non-pigmented skin) (p<0.05) (Figure 7A). However, the expression levels in liver tissues of the *mo^w^*/*mo^w^* and *mo*/*mo* chicks were similar to those of the wild type (Figure 7B).

## Discussion

In the present study, we identified a novel recessive mutation associated with white plumage in Minohiki and several other Japanese chicken breeds. Complementation tests revealed that the novel white plumage mutation was controlled by a *TYR* (*c*)-independent autosomal recessive gene at the mottled (*mo*) locus. This new mutant allele is designated *mo^w^* and is an allelic variant for *mo* of mottled chickens. Furthermore, the intergeneric hybrid between the white mutant chicken (*mo^w^*/*mo^w^*) and the *panda* mutant of Japanese quail (*s*/*s*) showed a *mo^w^*/*mo^w^* chicken-like plumage. This result showed that the mutations in parental species are alleles of the same gene, *EDNRB2*, which is the causative gene of the *panda* plumage of quail. Nucleotide sequencing revealed a non-synonymous nucleotide substitution from G to T at position 1,008 in exon 5 of *EDNRB2* in the white mutant chicken, which leads to the Cys244Phe amino acid substitution in EDNRB2. The association of this missense mutation (G1008T) with the white plumage phenotype was confirmed by genotyping of F_2_ progeny and several other Japanese chicken breeds with white plumage.

EDNRB2 is one of the receptors for EDNs, which are strong mitogens for melanoblasts [19], [20], [22], [23], [24]. Several types of EDNs and EDNRs are reported in a wide variety of vertebrates [32], [54], [55], which show molecular diversity in terms of gene duplication, mutation and chromosomal translocation during evolution [43]. *EDNRB2* is a paralog of *EDNRB* identified in birds, *Xenopus*, and platypus [24], [42], [43]. The avian EDNRB and EDNRB2 show similar pharmacological properties to EDN1, EDN2, and EDN3 [54]. The EDN3 and EDNRB signal transduction system is reported to be indispensable for the development of neural crest-derived melanocytes or enteric neurons [36], [37], [38]. Kawasaki-Nishihara et al. [24] suggested that EDN3–EDNRB2 signaling is required for normal melanoblast migration in *Xenopus* embryos on the basis of *in vivo* experiments. The present results indicated that the aberrant EDNRB2 causes hypopigmentation in feathers and suggested that normal EDNRB2 is essential for normal pigmentation in chickens, as in *Xenopus* or platypus [43].

**Figure 8:**
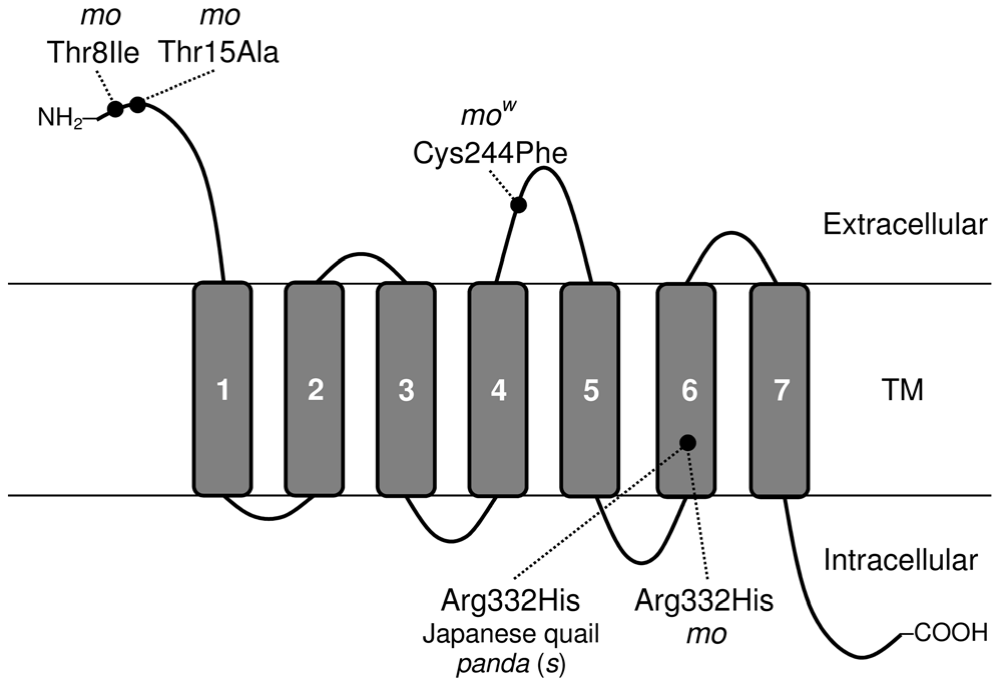
Schematic diagram of the structure of the EDNRB2 protein. Black circles indicate the positions of amino acid substitution in the *mo^w^* and *mo* alleles. The Cys244Phe substitution in the *mo^w^* allele was located in the extracellular loop between the putative fourth and fifth transmembrane (TM) domains of EDNRB2. The other three substitutions were identified at the *mo* locus: Thr8Ile and Thr15Ala in the N-terminal amino acids that constitute the cleaved peptide signal, and Arg332His in the sixth TM domain. The Arg332His substitution of EDNRB2 was previously reported in the *panda* (*s*/*s*) plumage mutant of Japanese quail (Miwa *et al*., 2007). The grey-shaded rectangles represent the TM domains.

The *mo^w^* mutation is located in the extracellular loop between the putative fourth and fifth transmembrane domains of EDNRB2 (Figure 8), which is considered to play an important role in the interaction with EDN3 [54]. Amino acid alignment of EDNRB and EDNRB2 shows that Cys-244 is evolutionally highly conserved in six species from four classes (Mammalia, Aves, Amphibia, and Osteichthyes) (Table 4). This result strongly suggests that the presence of Cys at position 244 is crucial for the basic function of EDNRB2. On the other hand, the Cys244Phe substitution was identified in five Japanese native breeds [Minohiki (Shizuoka), Onagadori, Ohiki, Shokoku, and Uzurao]. Except for Uzurao (a rumpless breed), all of these breeds have a long tail, and are considered to have a close genetic relationship [56], [57], [58]. This finding suggests that the *mo^w^* mutation may have originated from a common ancestor of a Japanese or Asian population of the long-tailed native chicken breeds. Therefore, the genetic analysis of plumage inheritance provides valuable information not only about the genetic and molecular basis of plumage colour but also the phylogenetic relationships and histories of chicken breeds.

We identified three mottled plumage-specific non-synonymous substitutions in the coding region of *EDNRB2* for four breeds (EJ, Chabo, CB, and Koshamo): two non-synonymous substitutions, Thr8Ile and Thr15Ala in the N-terminal putative signal peptide (1–23 residues) and Arg332His in the sixth transmembrane domain (SOSUIsignal, http://bp.nuap.nagoya-u.ac.jp/sosui/sosuisignal/) (Figure 8). These three mutations were found in all *mo* chickens that we analysed. Of the three mutation sites, Arg-332 is evolutionally highly conserved in *EDNRB* and *EDNRB2* of vertebrates. The Arg332His substitution was also identified in the *panda* plumage (*s*/*s*) mutant of Japanese quail [59]; however, the other mutations (Thr8Ile and Thr15Ala) were not found in the *panda* mutant quail. Interestingly, Thr8Ile and Thr15Ala mutations are also present in non-mottled chicken breeds (RIR, Large Cochin, and Sebright bantam). These results suggest that the Arg332His substitution is a primary cause of the mottled plumage pattern, although Thr8Ile and Thr15Ala are located in the region of the putative signal sequence (amino acids 1–23) of EDNRB2, which is generally considered to mediate the post-translational transport of the protein, these mutations may have little influence on binding to the ligand. However, these two non-synonymous mutations (Thr8Ile and Thr15Ala) may cause the difference in plumage pattern between the white mutant chicken and the *panda* quail, although the amino acid sequence homology of EDNRB2 is 97.7% (426/436 amino acids) between the wild-type chicken and Japanese quail. The phenotype of the *mo^w^* mutant chicken is similar to that of the *dotted-white* (*s^dw^*) mutant at the *s* (*EDNRB2*) locus in Japanese quail [49], [59]. No mutations were identified in the coding region of *EDNRB2* in this mutant quail; however, the expression level of the gene was reported to be remarkably declined compared with that of the wild type. This result suggests that the *s^dw^* mutation probably occurred in the regulatory region of the gene, thus leading to the lower quantity of EDNRB2. Chickens homozygous for the *mo^w^* allele showed much lower levels of pigmentation than those homozygous for the *mo* allele; nevertheless the *EDNRB2* expression level in liver tissue of *mo^w^* homozygotes was similar to that of the wild type. This result suggests that the Cys244Phe mutation in the *mo^w^* allele may not cause down-regulation of *EDNRB2* but may cause defective functioning of EDNRB2 with respect to binding with EDN ligands, thus resulting in interference with melanocyte differentiation, proliferation, and migration [32], [33], [34], [35]. To verify this function, we are now examining the gene expression in the developmental stage of embryos and in skins collected from all the shades of each feather color between the wild-type (*Mo* ^+^/*Mo* ^+^) and the mutant types (*mo*/*mo* and *mo^w^*/*mo^w^*).

Several outstanding questions remain to be addressed. Why did Aves acquire *EDNRB2* in addition to *EDNRB*? Do these receptors share other functions besides controlling pigmentation? Do these receptors show any differences in function or role in pigmentation? The functional divergence of these receptors and their molecular evolution are also interesting topics for future research. Mutation of *EDNRB* in mouse causes white coat colour and megacolon in the homologous condition, and these results in lethality within five weeks after birth [36], [39]. These abnormalities are considered to be caused by incomplete development of nerve ganglia because of abnormality of the EDN–EDNRB signal transduction system (reviewed in [60]). Given that such disorders are not observed in the chicken mutants, the EDN–EDNRB system appears to function normally in *mo^w^* and *mo* chickens. However, the dramatic effects of the *EDNRB2* mutation on pigment production in chickens and quails suggest that *EDNRB2* is required for avian melanoblasts to promote melanocyte differentiation and to enter the migration pathway from the neural crest. The EDN–EDNRB signal transduction system in mammals is understood to control the migration of nerve or bowel precursor cells by a dorsoventral route, and the migration of pigment cells mainly by a dorsolateral route. The avian *EDNRB* and *EDNRE2* genes may have evolved at partially different functions after the gene duplication; therefore, the EDN–EDNRB2 system in Aves is considered to play a part of the role of the EDN–EDNRB system, such as the differentiation of pigment cells. Fibromelanosis (*Fm*) of the Silkie chicken is a mutant phenotype of pigmentation, which exhibits extensive pigmentation of dermal layer of skin and internal connective tissues. This phenotype is caused by a duplication of a genomic region containing *EDN3*, which is a ligand of the EDN–EDNRB system and has a major role in melanocyte proliferation [25], [26]. To generate the birds with *mo^w^*/*mo^w^* and *Fm*/− genotype and to examine their phenotypes provide us a clue for understanding the function and molecular mechanism of the EDN–EDNRB2 signaling system in Aves. The *mo^w^* allele appears to be a malfunctional or loss-of-function mutation; therefore, the pigmentation is predicted to be modified or suppressed in the *mo^w^*/*mo^w^* background. We are now advancing this experiment. *EDNRB2*-mutated chickens with a variety of plumage patterns are an effective tool to elucidate the EDNRB2 function in the proliferation and differentiation of melanocytes and neural crest cells. In addition, such mutants are particularly important in order to analyse the molecular diversity of *EDNRB* in vertebrates and the origin of the *EDNRB2* mutation that occurred in Asian chicken breeds.

## Methods

### Allelism and progeny tests

For the allelism test of the white plumage mutant identified in the MH line, white MH females were mated with males of four tester lines: the Fayoumi (PNP/DO) (*i* ^+^/*i* ^+^, *Mo* ^+^/*Mo* ^+^, *C* ^+^/*C* ^+^); autosomal albino (CAL) (*i* ^+^/*i* ^+^, *Mo* ^+^/*Mo* ^+^, *c^a^*/*c^a^*); mottled Cochin bantam (CB) (*i* ^+^/*i* ^+^, *mo*/*mo*, *C* ^+^/*C* ^+^); and mottled Ehime-jidori (EJ) (*i* ^+^/*i* ^+^, *mo*/*mo*, *C* ^+^/*C* ^+^) lines (Table 1). We examined the colours of the chick down and adult plumage in F_1_, F_2_, and/or backcross progeny obtained from the four test matings. Following the mating of CAL with white MH, we examined the phenotypes and genotypes of the F_2_ progeny to determine the mode of inheritance for white plumage in the MH line, and performed linkage mapping of the white plumage locus. The segregation data of the F_2_ progeny were analysed with the chi-square test. Furthermore, To test the allelism of the white plumage (*mo^w^*/*mo^w^*) of chicken and the *panda* plumage (*s*/*s*) of Japanese quail whose causative gene is the *endothelin receptor B2* (*EDNRB2*) gene, an intergeneric F_1_ hybrid was obtained from artificially inseminated female *panda* quails with semen of the white chicken male. Animal care and all experimental procedures were approved by the Animal Experiment Committee, Graduate School of Bioagricultural Sciences, Nagoya University (approval nos 2012031206 and 2012050102), and the experiments were conducted according to Regulations on Animal Experiments at Nagoya University.

### cDNA sequencing of *EDNRB2*

We determined cDNA sequences of the entire coding region of *EDNRB2* for four individuals from MH and EJ lines (Table 2), which exhibited three different types of plumage: the wild-type (MH and EJ), white (MH), and mottled (EJ) plumage. Dorsal skin tissue (2.5 mm^2^) that contained developing feathers was dissected from one individual of each line and stored in RNAlater (Ambion, Austin, TX, USA). Total RNA was extracted using the TRIzol reagent (Invitrogen, Carlsbad, CA, USA) and 1.5 µg RNA was reverse-transcribed using the PrimeScript RT-PCR Kit (Takara, Otsu, Japan). The cDNA fragments including the entire coding region of *EDNRB2* were amplified using the primer pair EDNRB2_F1/EDNRB2_R1 (Figure S1; Table S1). Internal sequencing primers were used for direct sequencing, which were designed based on the cDNA sequence of chicken *EDNRB2* (GenBank accession no. NM_204120). PCR was carried out in a 50-µl reaction volume containing 50 ng cDNA, 1× KOD FX buffer, 200 µM dNTPs, 1.5 µl of 10 µM solution for each of the forward and reverse primers, and 1 unit KOD FX DNA polymerase (TOYOBO, Osaka, Japan). Each PCR reaction involved initial denaturation of 2 min at 94°C; 35 cycles of 98°C for 10 s, 64°C for 30 s, and 68°C for 2 min; and a final extension at 68°C for 7 min. The PCR products were purified from the gel using the Gel-M gel extraction kit (Viogene, Umeå, Sweden). Cycle-sequencing reactions used the Big Dye Terminator v3.1 Cycle Sequencing Kit (Applied Biosystems, Foster City, CA, USA) and nucleotide sequences were determined using an ABI PRISM 3130 DNA Analyser (Applied Biosystems).

### Linkage mapping

To confirm the association of the candidate gene (*EDNRB2*) with the white plumage mutation in MH line, we constructed a resource family by crossing a CAL male with a white MH female, as described above. Intercrossing of an F_1_ male with four F_1_ females generated 307 F_2_ progeny. Two microsatellite markers (*MCW0295* and *ADL0255*) on chromosome 4 were genotyped for the two grandparents, five F_1_ parents, and 93 F_2_ offspring. The PCR products of the markers were electrophoresed on either a 3.5% agarose gel or a 10% or 12% polyacrylamide gel. The genotyping of *EDNRB2* was performed 58 mutant individuals with white plumage and 184 individuals with the wild-type pigmented plumage excluding recessive albino individuals (n = 65). Linkage analysis between genotypes of microsatellite markers and *EDNRB2* and the plumage phenotypes was conducted using the Map Manager QTX software program [61]. Map units in centiMorgan (cM) were computed by applying the Kosambi function [62].

### Genomic DNA sequencing of *EDNRB2*

Genomic DNA sequences of exons 3 to 9 of the *EDNRB2* gene were determined for 18 chicken breeds including Red jungle fowl, which exhibited three different types of plumage (the wild-type, white and mottled plumage) (Table 3). Additionally, nucleotide sequences of genomic DNA fragments containing the open reading frame from exons 3 to 9 and their introns were determined for the wild-type MH, white MH mutant, and mottled EJ chickens. Blood samples were collected, and genomic DNA was extracted using DNAzol solution (Molecular Research Center, Cincinnati, OH, USA). The genomic fragment (5,914 bp) was amplified from 50 ng template genomic DNA using the primer pair EDNRB2_F1 and EDNRB2_R1 (Figure S1; Table S1). The internal primers were designed based on a reference genomic sequence on chromosome 4 in the UCSC chicken genome (May 2006 assembly). The PCR, which was carried out with KOD FX DNA polymerase (TOYOBO), involved initial denaturation of 2 min at 94°C; 35 cycles of 98°C for 10 s, 64°C for 30 s and 68°C for 6 min; and a final extension at 68°C for 7 min. The DNA sequence data sets were analysed using ATGC ver.5 sequence assembly software (Genetyx, Tokyo, Japan).

### Quantitative real-time RT-PCR

Expression of *EDNRB2* was analysed using quantitative real-time RT-PCR (qRT-PCR) for three-day-chicks with three different plumage phenotypes in three different genotypes: the wild type and white mutant chicks in the F_2_ progeny obtained from the mating of a white Shokoku male (*mo^w^*/*mo^w^*) with a GSP female (*Mo* ^+^/*Mo* ^+^); and the wild-type (*Mo* ^+^/*Mo* ^+^) and mottled (*mo*/*mo*) chicks in Ehime-jidori (EJ). Pigmented skin tissue of the wild-type chicks was collected from the back of the head, and pigmented and non-pigmented skin tissues were collected from the white mutant and mottled chicks. Total RNA was extracted from the head skin tissues including the pigmented and non-pigmented down feathers and liver tissues using the TRIzol reagent (Invitrogen), and complementary DNA was synthesised using the PrimeScript RT-PCR Kit (Takara). Quantitative real-time PCR was performed using SYBR *Premix Ex Taq*II (Takara) and the expressed dose was quantified with the StepOnePlus realtime PCR system (Applied Biosystems). The sequences of the primers were 5′-GCTTGCTTCATCCCGTTCAGA-3′ (sense) and 5′-ATGGCCAATGGCAAGCAGA-3′ (antisense). The following program conditions were applied for qRT-PCR analyses: 95°C for 30 s followed by 40 cycles that each comprised 95°C for 5 s followed by 60°C for 30 s following manufacturer's instruction. The expression levels were normalised against glyceraldehyde-3-phosphate dehydrogenase (*GAPDH*), which was amplified in the same run following the same procedure described above. The sequences of the *GAPDH* primers used were 5′–GGAGAAACCAGCCAAGTATGATG–3′ (sense) and 5′–AAAGGTGGAGGAATGGCTGTCA–3′ (antisense). Each quantitative PCR was performed in triplicate to verify the results, and the mean mRNA expression was used for data analysis. Gene expression level was analysed using the 2^−ΔΔCT^ method. Expression levels in skin and liver tissues were statistically compared among experimental groups by one-way ANOVA (analysis of variance) followed by Tukey HSD (honestly significant difference) test of the statistical discovery software JMP version 10.0.2 (SAS Institute, Cary, NC).

## Supporting Information

Figure S1 **Genomic structure of** ***EDNRB2*** **.** Arrows indicate the PCR primers that were used for nucleotide sequencing. The primers with asterisks were used for genotyping of the G1008T (Cys244Phe) and G1272A (Arg322His) mutations. White boxes indicate untranslated exons at 5′ and 3′ ends, and black boxes indicate the coding exons.
(DOCX)

Figure S2 **Genetic linkage map of the** ***mo^w^*** **mutation on chicken chromosome 4.** The linkage map was constructed using a population of 93 F_2_ progeny (50 wild-type and 43 white mutant individuals) obtained from the mating of a CAL male with a white MH female. Two microsatellite markers (*ADL0255* and *MCW0295*) and *EDNRB2* on chromosome 4 were genotyped for the F_2_ progeny, and their genotypes were compared with the segregation patterns of plumage.
(DOCX)

Figure S3 **Partial sequences in intron 5 of** ***EDNRB2*** **in the Minohiki (MH) line and the mottled Ehime-jidori (EJ) line.** (A) The 432-bp LTR/ERVK sequence is located at positions 11,261,544–11, 261,975 of intron 5 on the basis of the chicken reference genomic sequence (Type I). This LTR/ERVK sequence is absent in intron 5 of *EDNRB2* in the MH line (*Mo* ^+^/*Mo* ^+^, *mo^w^*/*mo^w^*) and the mottled EJ line (*Mo* ^+^/*Mo* ^+^, *mo*/*mo*) (Type II), in which the unrelated 295-bp sequence replaced the LTR/ERVK at the same position. (B) Comparison of partial sequences in intron 5 of Type I and II sequences.
(DOCX)

Table S1 **List of primers used for amplifying DNA fragments of chicken.**
(XLSX)

Table S2 **Distribution of the G1008T nucleotide substitution leading to Cys244Phe in** ***EDNRB2*** **in 22 chicken breeds including Red jungle fowl.**
(XLSX)

Table S3 **Distribution of the G1272A nucleotide substitution leading to Arg332His in** ***EDNRB2*** **in 14 chicken breeds including Red jungle fowl.**
(XLSX)

Table S4 **DNA sequence polymorphism in** ***EDNRB2*** **among three alleles (** ***Mo*** **^+^,** ***mo^w^*** **and** ***mo*** **).**
(XLSX)
