# Supplementary material for: Endothelin Receptor B2 (EDNRB2) Is Responsible for the Tyrosinase-Independent Recessive White (mow) and Mottled (mo) Plumage Phenotypes in the Chicken
Source: PLoS One. 2014 Jan 23;9(1):e86361. doi: 10.1371/journal.pone.0086361 (PMC3900529; doi:10.1371/journal.pone.0086361)
Supplement: Figure S1 — Genomic structure of EDNRB2 . Arrows indicate the PCR primers that were used for nucleotide sequencing. The primers with asterisks were used for genotyping of the G1008T (Cys244Phe) and G1272A (Arg322His) mutations. White boxes indicate untranslated exons at 5′ and 3′ ends, and black boxes indicate the coding exons. (DOCX) [file pone.0086361.s001.docx]

Int2F

*

*

Int6F

Int5F1

F1

Int3F1

Int3F2

Int3F

Int4F

E4

E2

E7

E6

E5

E8

E9

E3

E1

RS2

RS3

RS1

Int6R

R1

Ex8R

Int5R2

Int5R1

Int3R

*

*

Int8R1

Int8R2

500 bp
