# Supplementary material for: Endothelin Receptor B2 (EDNRB2) Is Responsible for the Tyrosinase-Independent Recessive White (mow) and Mottled (mo) Plumage Phenotypes in the Chicken
Source: PLoS One. 2014 Jan 23;9(1):e86361. doi: 10.1371/journal.pone.0086361 (PMC3900529; doi:10.1371/journal.pone.0086361)
Supplement: Figure S2 — Genetic linkage map of the mow mutation on chicken chromosome 4. The linkage map was constructed using a population of 93 F2 progeny (50 wild-type and 43 white mutant individuals) obtained from the mating of a CAL male with a white MH female. Two microsatellite markers (ADL0255 and MCW0295) and EDNRB2 on chromosome 4 were genotyped for the F2 progeny, and their genotypes were compared with the segregation patterns of plumage. (DOCX) [file pone.0086361.s002.docx]

Linkage map

(cM)

*ADL0255*

41.1

20.2

GGA4

(Mb)

*ADL0255*

2.2

*EDNRB2*

11.39.0

*MCW0295*

16.3

*EDNRB2*/***mo*** (***mo^w^***)

*MCW0295*
