# Supplementary material for: Endothelin Receptor B2 (EDNRB2) Is Responsible for the Tyrosinase-Independent Recessive White (mow) and Mottled (mo) Plumage Phenotypes in the Chicken
Source: PLoS One. 2014 Jan 23;9(1):e86361. doi: 10.1371/journal.pone.0086361 (PMC3900529; doi:10.1371/journal.pone.0086361)
Supplement: Figure S3 — Partial sequences in intron 5 of EDNRB2 in the Minohiki (MH) line and the mottled Ehime-jidori (EJ) line. (A) The 432-bp LTR/ERVK sequence is located at positions 11,261,544–11, 261,975 of intron 5 on the basis of the chicken reference genomic sequence (Type I). This LTR/ERVK sequence is absent in intron 5 of EDNRB2 in the MH line (Mo +/Mo +, mow/mow) and the mottled EJ line (Mo +/Mo +, mo/mo) (Type II), in which the unrelated 295-bp sequence replaced the LTR/ERVK at the same position. (B) Comparison of partial sequences in intron 5 of Type I and II sequences. (DOCX) [file pone.0086361.s003.docx]

**A**

E1

E2

E3

E4

E5

E6

E7

E8

E9

Exon 6

Intron 5

432 bp

I

Exon 5

11261224　 11261543 11261976　11262033

LTR/ERVK

295 bp

II

Exon 5

Exon 6

11261224　 11261543 11261976　11262033

**B**

I

II

AGGCTGTCAGAACAGGCCAAGGAGTGTTGCATGTAGTCTCCGTTCGCTCGTCCGGTGTTC

AGGCTGTCAGAACAGGCCAAGGAGCATC--------------------------------

************************ *

11261543

I

II

GTCCTCTGTCCACATGTAGGGCTTACTGCTGGGCGAAACCGACCCTTTACCAGGTCGGGG

--------TCCACAACCAG-----ACTGC-------------------ACAAGGCCAG--

****** ** ***** ** *** * *

I

II

CCAGATGCTCACCCAGACCCCAGGAGTAAGTGAGGCAAATGGCGTTTATTGCTATAGGCT

-------------------CTAAGGGTGGGCAAGG-AGATGAAGGCCTTTGCAAAGCATC

* * * ** * *** * *** * **** *

I

II

ACGTGTTTAAATACAAGTGTTTCCTCCAATCACGAAGTTACACTTGGCACACACAGGTGG

AC-------------AACTTGCCTTCTCACCCCAAGTCT-------GCCTAAATAAGCAC

** * * * ** * * * * * ** * * * *

I

II

CATAACACACAGGTGGCATAGGAACCTGCACGCGTCACACCTCGTTTCCCTCGCTACGCC

CTTGCAAC-CATTTCTTGTACAATCTTGCTAGATTGGCA-------------GCCAGGTG

* * ** ** * ** * * *** * * ** ** * *

I

II

TACAACACACCTCGTTTCCCTCGCTACGCATACAACATCTCCCCCTCCCTATGCCGATAG

TAAACCAGACTTCCCCTCTTTGG-------------GTTAAGCCCTGGGGGGGACGATGA

** * ** ** ** ** * * * **** * ****

I

II

CTCCCATCACATCGCGTTCGGAACCTACAGCTGCTCCCTAATTCTATGATATTTTATGCG

TCCCATGTACACTTAGCCCAGGGGCTGTTACACTTTCGTACATCAGAAGTGTCAGAGAGG

** ** * * * ** * * * ** ** * * * *

I

II

TTCATCCATAAACTGATTCATTGTTGTCAGGGACAAATGCATCAGCTACCTTCCTTCTTC

TCTG---ATAAGTGACAGCACTGGTGTCAGGGACCAATGCATCAGCTACCTTCCTTCTTC

* **** ** ** ********** *************************

11261976
